# Graphene-Based Ion-Selective Field-Effect Transistor for Sodium Sensing

**DOI:** 10.3390/nano12152620

**Published:** 2022-07-29

**Authors:** Ting Huang, Kan Kan Yeung, Jingwei Li, Honglin Sun, Md Masruck Alam, Zhaoli Gao

**Affiliations:** 1 Biomedical Engineering Department, The Chinese University of Hong Kong, Shatin, New Territories, Hong Kong, China; irishhh@link.cuhk.edu.hk (T.H.); kyeung@cuhk.edu.hk (K.K.Y.); jlidt@connect.ust.hk (J.L.); sunhl20@link.cuhk.edu.hk (H.S.); masruckalam@link.cuhk.edu.hk (M.M.A.); 2 CUHK Shenzhen Research Institute, Nanshan, Shenzhen 518057, China; 3 Department of Chemical and Biological Engineering, The Hong Kong University of Science and Technology, Clear Water Bay, Kowloon, Hong Kong, China

**Keywords:** ion-selective field-effect transistor, graphene, sodium ions, real-time monitoring

## Abstract

Field-effect transistors have attracted significant attention in chemical sensing and clinical diagnosis, due to their high sensitivity and label-free operation. Through a scalable photolithographic process in this study, we fabricated graphene-based ion-sensitive field-effect transistor (ISFET) arrays that can continuously monitor sodium ions in real-time. As the sodium ion concentration increased, the current–gate voltage characteristic curves shifted towards the negative direction, showing that sodium ions were captured and could be detected over a wide concentration range, from 10^−8^ to 10^−1^ M, with a sensitivity of 152.4 mV/dec. Time-dependent measurements and interfering experiments were conducted to validate the real-time measurements and the highly specific detection capability of our sensor. Our graphene ISFETs (G-ISFET) not only showed a fast response, but also exhibited remarkable selectivity against interference ions, including Ca^2+^, K^+^, Mg^2+^ and NH_4_^+^. The scalability, high sensitivity and selectivity synergistically make our G-ISFET a promising platform for sodium sensing in health monitoring.

## 1. Introduction

Sodium ions are important indicators for monitoring and evaluating health status owing to their important role in homeostasis and maintaining the proper functions of the nervous system [1,2,3]. For instance, the total sodium level in cognitively normal brain tissues is around 35–45 mM, and 12–21 mM in healthy muscle tissue [4,5,6]. Deviation of sodium concentrations in the human body is related to its hydration status, which can be used as an indicator for health monitoring [7,8]. Thus, rapid, reliable and real-time monitoring of sodium ions has been an increasing interest in the fields of precision medicine and personalized healthcare [1,9,10]. To date, solid-contact ion-selective electrodes (ISE) are the most commonly used platforms for ion sensing, due to their low cost, accuracy, and simple operation [11,12,13,14,15]. However, ISEs have drawbacks, including the relatively high detection limit and narrow detection range, e.g., 10^−4^ or 10^−5^ M for specific ions [16,17,18,19].

Recently, field-effect transistors (FET) have gained increasing attention in ion sensing, offering the prospect of simple, rapid, cost-effective, and label-free detection [20,21]. The FET biosensors hold tremendous promise for label-free detection of target molecules with high accuracy and selectivity, without the usage of fluorescent, isotopic, or electrochemical labeling [22,23]. In combination with an ion-selective membranes (ISM), ion-sensitive field-effect transistors (ISFETs) are promising for ion sensing with enhanced sensitivity, and reduced sensor sizes and response times, providing the possibility to integrate them with flexible electronics [24,25,26,27,28].

Graphene is a 2D material with unique material properties, such as high carrier mobility (up to 10^6^ cm^2^/V·s) [29], high conductivity [30], excellent mechanical strength, etc. [30,31]. Taking advantage of all these features combined, we have fabricated G-ISFETs that offer high sensitivity, selectivity and real-time monitoring of sodium ions. The graphene channel was grown by atmospheric pressure chemical vapor deposition (CVD), and transferred to pre-patterned electrodes, followed by a scalable photolithographic process. The graphene FETs (GFETs) were then functionalized with a sodium ionophore to specifically capture the target sodium ions. A broad range of sodium concentrations, from 10^−8^ to 10^−1^ M, which covers the sodium concentration in tissues, was detected, with a sensitivity of 152.4 mV/dec. We further conducted time-dependent measurements and control experiments to demonstrate the capability of real-time monitoring with high selectivity. The high performance of our G-ISFET makes it a promising platform for the real-time monitoring of sodium ions for health monitoring through physiological liquids.

## 2. Materials and Methods

### 2.1. Graphene Synthesis

The monolayer graphene film was synthesized using a chemical vapor deposition system (Lindberg/Blue M™ Mini-Mite™ Thermo Scientific Co., Waltham, MA, USA). The copper foil (Alfa Aesar, #13382, Haverhill, MA, USA) was cleaned by sonication in 5.4% HNO_3_ for 1 min and then rinsed in DI water twice, followed by drying with high-pressure nitrogen gas. The cleaned foil was then transferred into the quartz tube. The furnace was heated to 1050 °C with a constant flow of 500 sccm Ar and 30 sccm H_2_ and then annealed for 5 min. The 5 sccm-diluted CH_4_ (0.5% in Ar) was introduced as a carbon source, and the growth time was 1 h. Lastly, the furnace was rapidly cooled to room temperature under the H_2_ and Ar atmosphere.

### 2.2. GFET Sensor Array Fabrication

The sensor fabrication process was summarized in Figure S1. First, the electrode pattern was defined on a 4-inch p-doped SiO_2_ (285 nm)/Si wafer by standard photolithography. The contact metallization was 8 nm Cr/45 nm Au, deposited by e-beam evaporation. Monolayer graphene was then transferred onto the pre-patterned SiO_2_/Si chip using a “bubbling” transfer method. Briefly, a layer of polymethylmethacrylate (PMMA) was spin-coated on the graphene-Cu foil, followed by baking at 105 °C for 2 min and then slowly immersed into a 50 mM NaOH aqueous solution [32]. By applying a 15 V voltage, the graphene/PMMA film was peeled off from Cu foil by the hydrogen bubbles formed on the copper surface. The film was washed with DI water thrice and transferred onto the electrode chip. The chip was air-dried and then baked at 150 °C for 2 min before removing the PMMA with acetone. The graphene/electrode chip was then spin-coated with PMGI (Micro Chem Corp., Newton, MA, USA) and a S1813 (Shipley) photoresist bilayer and exposed using an ABM aligner. Graphene outside the channels was removed by O_2_ plasma etching. The remaining photoresist on graphene channels was stripped by Remover PG (Micro Chem Corp., Newton, MA, USA), acetone, and IPA. Finally, the GFET arrays were annealed in Ar/H_2_ forming gas at 225 °C to remove photoresist residues.

### 2.3. Ionophore Membrane Preparation

Selectophore grade sodium ionophore X (4-tertbutylcalix [4]arene-tetraacetic acid tetraethyl ester), sodium tetrakis [3,5-bis(trifluoromethyl) phenyl] borate (Na-TFPB), 2-nitrophenyl octyl ether (2-NPOE), tetrahydrofuran (THF), and poly (vinyl chloride) (PVC) were purchased from Sigma-Aldrich. The ionophore membrane was prepared by mixing 1 mg sodium ionophore X, 47.2 mg PVC, 90.7 µL 2-NPOE, and 0.29 mg Na-TFPB [33]. The mixture was dissolved in 1 mL THF and sonicated for 1 h, then stored at 4 °C for further usage.

### 2.4. Material Characterization

Micro-Raman measurements were performed by using WiTec Alpha 300 system with a laser excitation wavelength of 532 nm. An atomic force microscope (AFM, Icon Bruker, Tucson, AZ, USA) was used to characterize the height increase during the fabrication process.

### 2.5. Solution Preparation

Sodium chloride (NaCl), potassium chloride (KCl), magnesium chloride (MgCl_2_), calcium chloride (CaCl_2_) and ammonium chloride (NH_4_Cl) anhydrous salts with >99% purity were obtained from Sigma Aldrich. The desired concentrations were carefully prepared and diluted with de-ionized water (18.2 MΩ cm, Milli-Q^®^ 3 UV Water Purification System). The sweat sample was collected from a cycling volunteer at different sporting times, and stored in −20 °C refrigerator before testing.

### 2.6. Electrical Measurement

The 285 nm-thick SiO_2_ served as the gate dielectric, and the highly p-doped silicon substrate acted as the back-gate electrode. No liquid gate was applied in this study. The I-V_g_ characteristic measurements were performed after each functionalization step. The probe station (FormFactor MPS 150, Livermore, CA, USA) was equipped with a customized probe card, allowing 100 devices to be measured simultaneously. The Keithley 2400 source meter was used to apply a bias voltage (V = 0.1 V), and the gate voltage was applied using the Keithley 6517 model. A Python program was developed to conduct the measurement and collect data.

## 3. Results and Discussion

**Figure 1:**
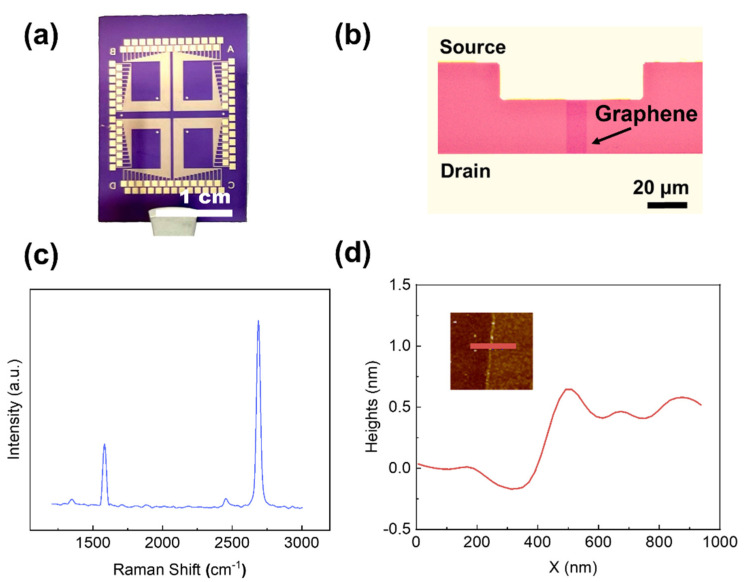
(**a**) Optical image of as-fabricated GFETs, (**b**) Optical image of the graphene channel and source/drain electrode. (**c**) Raman spectrum of a graphene channel after the fabrication process. Two characteristic peaks were found: G peak at ~1580 cm^−^^1^ and 2D peak at ~2700 cm^−1^ (**d**) The line scan profile of the as-annealed GFET, Inset: AFM image with scan line indicated. The thickness of the graphene channel is ~0.5 nm.

Figure 1a shows an optical image of a GFET fabricated by the photolithographic process. The monolayer graphene film was synthesized on a copper foil using chemical vapor deposition, followed by a hydrolysis bubble transfer onto a SiO_2_/Si chip with prefabricated Cr/Au electrodes to create an array of 100 GFETs. The graphene channel, as shown in Figure 1b, was defined by photolithography and oxygen plasma etching. The GFET chip was then annealed in an Ar/H_2_ atmosphere to remove any photoresist residues on the graphene channels [34]. The high quality of the as-fabricated GFETs was verified by the negligible D peak (~1345 cm^−1^) in the Raman spectrum (Figure 1c) [35]. The height of the GFET channel was ~0.5 nm, and there was a ~5 μm height increase after the immobilization of the sodium ionophore membrane. The Raman spectrum and AFM image together confirm the high quality of the as-grown CVD graphene, even after the photolithographic process.

**Figure 2:**
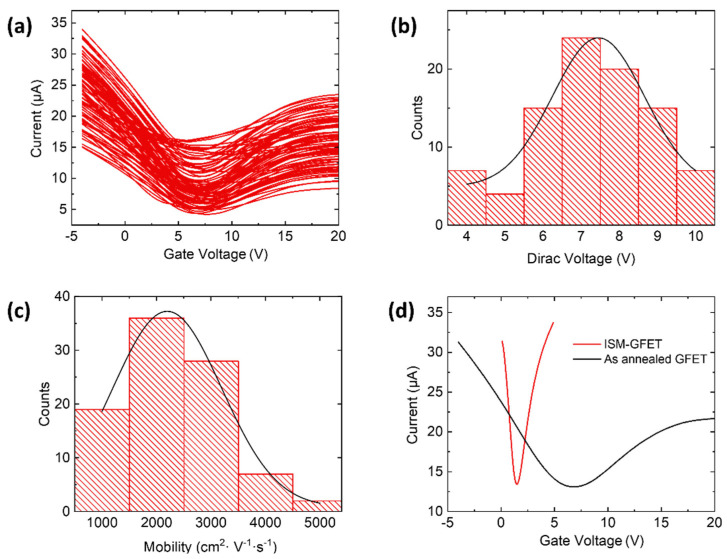
(**a**) Current-gate voltage characteristic of an array of 100 graphene field-effect transistors. Histograms and Gaussian fits (black lines) of (**b**) Dirac voltage and (**c**) hole mobility extracted from the curves in panel **a**. (**d**) Current-gate voltage characteristic curves before and after ionophore deposition.

As seen in Figure 2, the current-back gate voltage (I-V_g_) measurements show good device-to-device uniformity across the 100 arrays. The Dirac voltage and carrier mobility were extracted by fitting the hole branch of the I-V_g_ curve to the following equation [36,37]:

(1) $$\sigma^{-1}\left(V_{g}\right)\;={\left[\mu c_{g}\left(V_{D}-V_{g}\right)\right]}^{-1}+\sigma_{s}^{-1}$$

where *c_g_* is the gate capacitance per unit area (12.1 nF cm^−2^ for the 285 nm thick SiO_2_), *μ* is the hole carrier mobility, $\sigma_{s}$ is the saturation conductivity when *V_g_* approaches −∞. The narrow distribution of the Dirac point voltage (6.3 ± 4 V) and hole carrier mobility (2400 ± 600 cm^2^ V^−1^ s^−1^) indicates a low doping effect induced by the fabrication process.

**Figure 3:**
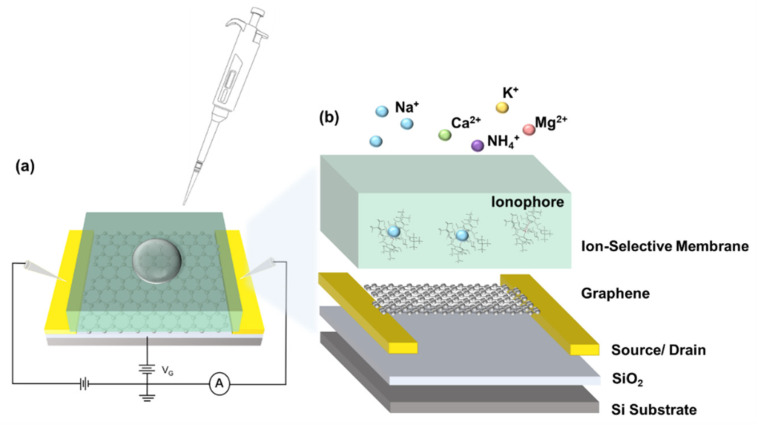
(**a**) Schematic of a back-gated G-ISFET. (**b**) Schematic of ionophore-functionalized GFET. The sodium ions captured in ionophores lead to a doping effect of the GFET.

The as-fabricated GFETs were then functionalized with the prepared sodium selective membrane, as shown in Figure 3. Briefly, the sodium ionophore X was dissolved and mixed with ion-selective membrane (ISM) cocktails (see Materials and Methods). An amount of 25 µL of the solution was drop-cast on the GFET surface, followed by air-drying overnight, to obtain the G-ISFET. The I-V_g_ characteristics was measured after the ionophore deposition, where the deposition of the ionophore leads to a negative Dirac point shift (Figure 2d). During sensing, the intrinsic structure of ionophore X, namely the calix [4] arenes, provides a scaffold with an optimum cavity for the complexation of sodium ions [38,39]. The captured ion in the sodium-selective membrane resulted in a surface potential change and the Dirac voltage shift in the characteristics curve.

**Figure 4:**
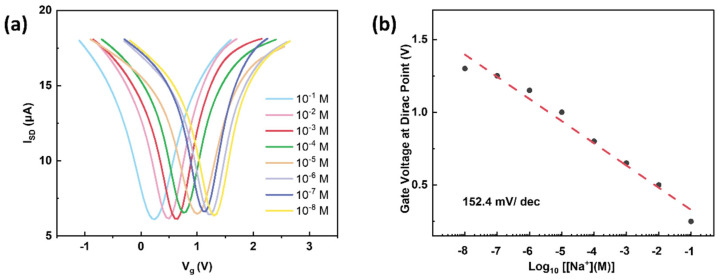
(**a**) Transport characteristic curves of G-ISFET against different Na^+^ concentrations from 10^−8^ to 10^−1^ M with a bias voltage of 100 mV. (**b**) G-ISFET response as a function for different target sodium concentrations at the logarithmic scale. A response of 152.4 mV/dec was observed.

A real-time measurement of the drain-source current through the ISM without the graphene channel against different sodium solutions (10^−5^, 10^−3^, and 10^−1^ M) was conducted, as shown in Figure S2, and the leaking current between the source and drain electrodes was found at the sub-nA level, which did not affect our study. The G-ISFET was tested against a series of sodium concentrations, from 10^−8^ to 10^−1^ M, to confirm the sensor response. The ion sensitive membrane provided a cation exchange site and created a barrier that prevented nonspecific ions from reaching the sensing surface. As a result, only sodium ions were able to permeate and pass through the selective membrane to reach the ISM–graphene interface. Accordingly, the sodium ion accumulation on the graphene surface caused a doping effect. This G-ISFET response is shown in Figure 4. A fixed bias voltage of 100 mV was applied during the sensing measurements. As the sodium concentrations increased, there was a consistent trend of negative shifts in the transport curves. This Dirac point shift was attributed to the increase in the electron concentration on the graphene’s surface, due to the accumulation of positively charged Na^+^ ions, thereby driving the Fermi level closer to the charge neutrality point through chemical gating, and consequently decreasing the Dirac point. The dependence of V_D_ on varying Na^+^ values is plotted in Figure 4b, where the dotted line represents a linear fit. The slope of calibration fitting reflects the sensitivity of the G-ISFET, i.e., 152.4 mV/dec. The sensitivity is comparable to that of recent reports (see Table S1) [20,36,40], presumably attributed to the atomically thin nature of the graphene and the scalable fabrication of high-quality sensor arrays based on CVD graphene.

**Figure 5:**
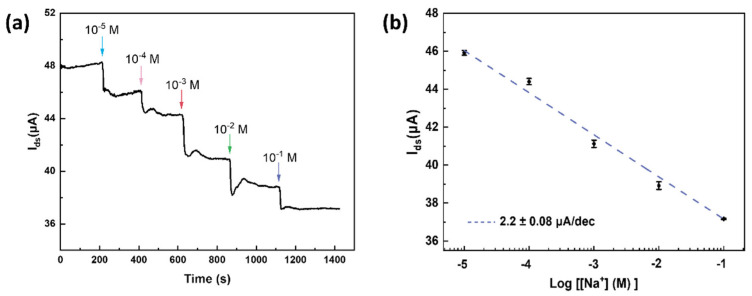
(**a**) Real-time response of the source–drain current against a series of Na^+^ molar concentrations (V_g_ = 0 V). (**b**) The linear response in I_DS_ with different sodium concentrations from the real–time measurements in panel a.

We next investigated the real-time response of G-ISFET against various sodium concentrations, by measuring I_DS_ versus sensing time with a fixed gate voltage (V_ds_ = 100 mV). As shown in Figure 5a, the source-drain current decreased with the increasing Na^+^ concentration, in agreement with the n-doping effect by positively charged Na^+^ ions. The linear response in I_DS_ is plotted in Figure 5b, and the fitting indicates a response of 2.2 ± 0.08 μA/dec, consistent with previously reported ISFETs [41,42,43].

**Figure 6:**
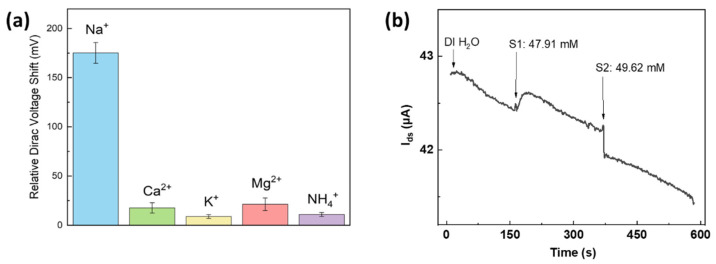
(**a**) The selectivity test against several interfering ions. Error bars are the standard deviation of the mean. (**b**) Real-time responses of G-ISFET against human sweat samples collected from a sporting volunteer.

Selectivity is a crucial factor in evaluating the performance of an ion sensor. We further carried out interference experiments to verify the effectiveness of our G-ISFET. As shown in Figure 6, several non-specific ions were tested, including Ca^2+^, K^+^, Mg^2+^ and NH_4_^+^, and the relative Dirac point shift was plotted. In sharp contrast to the large Dirac voltage shift for sodium ions, the as-fabricated G-ISFET displays a negligible response to the interfering ions, indicating that the ion-selective membrane specifically captured the target ions, and possessed excellent selectivity against nonspecific ions. We also performed measurements with a real sample, i.e., human sweat. As shown in Figure 6b, the source-drain current decreased with the increasing concentration of sodium ions (from 47.91 mM to 49.62 mM). This result confirmed the high selectivity and rapid response of the G-ISFET, which offers a pathway toward health evaluation through sweat.

## 4. Conclusions

We developed a graphene-based ISFET, incorporated with an ion-selective membrane, that can selectively detect sodium ions with high sensitivity. We grew large-area, high quality monolayer graphene by chemical vapor deposition, followed by a scalable photolithographic process, to fabricate the GFETs. The as-fabricated GFETs were then functionalized with sodium ionophore to sensitively capture sodium ions. We detected sodium ions with a wide range of concentrations, from 10^−8^ to 10^−1^ M, and achieved a sensitivity of 152.4 mV/dec, comparable to previously reported ISFET sensors. Nevertheless, the back-gate architecture of G-ISFET eliminates the usage of reference electrodes, offering a way to miniaturize the ISFET device. We further conducted time-dependent measurements and interfering experiments to demonstrate the real-time response and selectivity capabilities of our G-ISFETs, showing a fast response to changes in concentration, and exhibiting excellent selectivity against interference ions, including Ca^2+^, K^+^, Mg^2+^ and NH_4_^+^. The scalability, sensitivity and selectivity synergistically make our G-ISFET a promising candidate for sodium sensing in health monitoring.

## Data Availability

Not applicable.

## Supplementary Materials

The following supporting information can be downloaded at: https://www.mdpi.com/article/10.3390/nano12152620/s1, Figure S1: Illustration of the scalable fabrication process of G-ISFET; Figure S2: Real-time measurement of drain-source current with a bare ISM; Table S1: Comparison of ion sensitivities for sodium sensing.
