# Supplementary material for: Graphene-Based Ion-Selective Field-Effect Transistor for Sodium Sensing
Source: Nanomaterials (Basel). 2022 Jul 29;12(15):2620. doi: 10.3390/nano12152620 (PMC9370261; doi:10.3390/nano12152620)
Supplement: Supplementary file 1 [file nanomaterials-12-02620-s001.zip › nanomaterials-1820073-supplementary.pdf]

nanomaterials MDPI

Supplementary Materials

Graphene-Based Ion-Selective Field-Effect Transistor for Sodium Sensing

Ting Huang [1], Kan Kan Yeung [1,2], Jingwei Li [1,3], Honglin Sun [1], Md Masruck Alam [1] and Zhaoli Gao [1,2,*]

[1] Biomedical Engineering Department, The Chinese University of Hong Kong, Shatin, New Territories, Hong Kong, China; irishhh@link.cuhk.edu.hk (T.H.); kyeung@cuhk.edu.hk (K.K.Y.); jlidt@connect.ust.hk (J.L.); sunhl20@link.cuhk.edu.hk (H.S.); masruckalam@link.cuhk.edu.hk (M.M.A.)
[2] CUHK Shenzhen Research Institute, Nanshan, Shenzhen 518057, China
[3] Department of Chemical and Biological Engineering, The Hong Kong University of Science and Technology, Clear Water Bay, Kowloon, Hong Kong, China.
* Correspondence: zlgao@cuhk.edu.hk

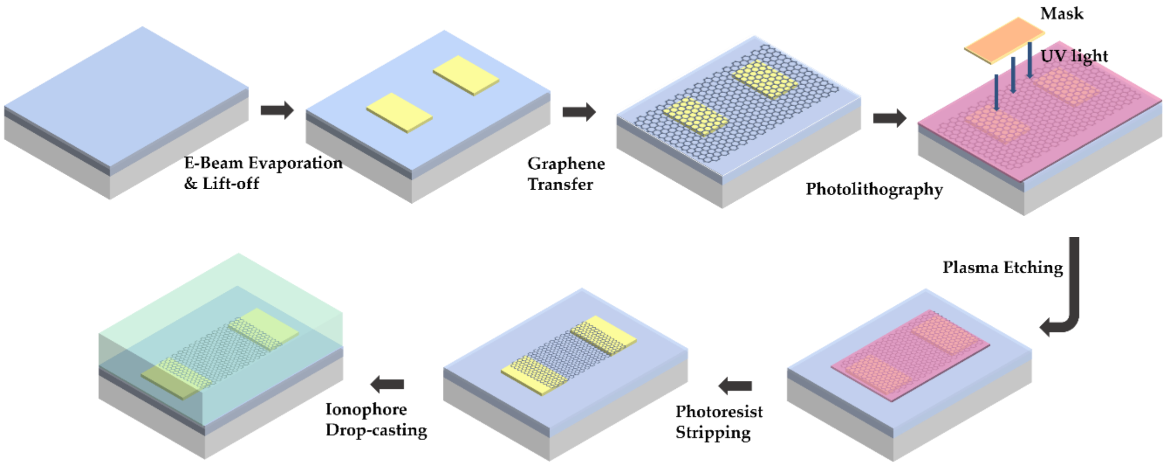

Figure S1. Schematic of the scalable fabrication process of the G-ISFETs. Graphene was grown via atmosphere pressure CVD on a Cu foil and wet-transferred to the pre-patterned SiO_2/Si wafer using a PMMA-assisted bubbling transfer method. The graphene channel was defined by a photolithography process and etched through a plasma etcher, followed by stripping of the remaining photoresist. The prepared sodium Ionophore was applied by drop-casting.

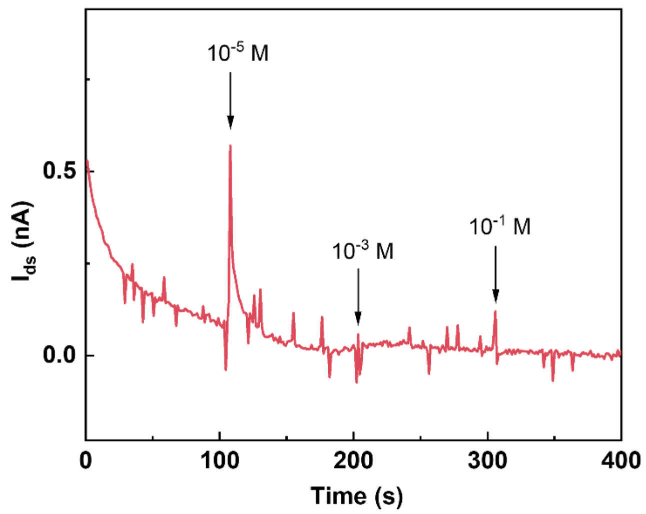

Figure S2. Real-time measurement of drain-source current with a bare ISM, against varying sodium concentrations (10^{-5} M, 10^{-3} M, and 10^{-1} M). The drain-source current is at the nA level, three orders of magnitude lower than the G-ISFET. This result indicates that the leaking current through the sodium solutions was negligible in our study.

Table S1. Comparison of ion sensitivities for sodium sensing.

Type	Sensing channel	Detection Range	Sensitivity	Ref
ISFET	silicon nanowire (SiNW)	10^{-4} –10^{0} M	1464.66 mV/dec	[1]
ISFET	Carbon Nanotube (CNT)	10^{-4} –10^{-1} M	71.7 mV/dec	[2]
OFET	Poly(3-hexylthiophene)	10^{-6}–10^{-1} M	62 mV/dec	[3]
FinFET	Al_2O_3 Nanoseaweed	NA	432.7 mV/ dec	[4]
ISFET	Graphene	10^{-4} – 10^{0} M	55.4 mV/dec	[5]
ISFET	Graphene	10^{-5} – 10^{-1} M	49.2 mV/ dec	[6]
ISFET	Graphene, Graphene/ hBN	10^{-4} – 10^{0} M	135, 160 mV/ dec	[7]
ISFET	Graphene	10^{-8} – 10^{-1} M	152.4 mV/ dec	This work

Reference

1. Cho, S.K. and W.J. Cho, *Highly Sensitive and Selective Sodium Ion Sensor Based on Silicon Nanowire Dual Gate Field-Effect Transistor.* Sensors, 2021. **21**(12).
2. Park, S.-C., et al., *Carbon Nanotube-Based Ion-Sensitive Field-Effect Transistors with an On-Chip Reference Electrode Toward Wearable Sodium Sensing.* ACS Applied Electronic Materials, 2021. **3**(6): p. 2580-2588.
3. Schmoltner, K., et al., *Electrolyte-Gated Organic Field-Effect Transistor for Selective Reversible Ion Detection.* Advanced Materials, 2013. **25**(47): p. 6895-6899.
4. Shen, Y.C., et al., *Multifunctional Ion-Sensitive Floating Gate Fin Field-Effect Transistor with Three-Dimensional Nanoseaweed Structure by Glancing Angle Deposition Technology.* Small, 2022. **18**(5).
5. Oh, H.G., et al., *Two-Dimensional Disposable Graphene Sensor to Detect Na+ Ions.* Nanomaterials, 2021. **11**(3).
6. Fakih, I., et al., *Selective ion sensing with high resolution large area graphene field effect transistor arrays.* Nat Commun, 2020. **11**(1): p. 3226.
7. Hasan, N., et al., *Ion-Selective Membrane-Coated Graphene–Hexagonal Boron Nitride Heterostructures for Field-Effect Ion Sensing.* ACS omega, 2021. **6**(45): p. 30281-30291.
